# Gastric cancer mesenchymal stem cells regulate PD-L1-CTCF enhancing cancer stem cell-like properties and tumorigenesis

**DOI:** 10.7150/thno.49717

**Published:** 2020-10-25

**Authors:** Li Sun, Chao Huang, Miaolin Zhu, Shuwei Guo, Qiuzhi Gao, Qianqian Wang, Bin Chen, Rong Li, Yuanyuan Zhao, Mei Wang, Zhihong Chen, Bo Shen, Wei Zhu

**Affiliations:** 1 School of Medicine, Jiangsu University, Zhenjiang, Jiangsu, China; 2 Department of Clinical Laboratory, Kunshan First People's Hospital, Affiliated to Jiangsu University, Kunshan, Jiangsu, China; 3 Department of Oncology, Jiangsu Cancer Hospital Affiliated to Nanjing Medical University, Nanjing, Jiangsu, China; 4 Department of Gastrointestinal Surgery, Affiliated People's Hospital of Jiangsu University, Zhenjiang, Jiangsu, China

**Keywords:** gastric cancer, mesenchymal stem cell, cancer stem cell, PD-L1, CTCF.

## Abstract

**Rationale:** Mesenchymal stem cells (MSCs) have been the focus of many studies because of their abilities to modulate immune responses, angiogenesis, and promote tumor growth and metastasis. Our previous work showed that gastric cancer MSCs (GCMSCs) promoted immune escape by secreting of IL-8, which induced programmed cell death ligand 1 (PD-L1) expression in GC cells. Mounting evidence has revealed that PD-L1 expression is related to intrinsic tumor cell properties. Here, we investigated whether GCMSCs maintained a pool of cancer stem cells (CSCs) through PD-L1 signaling and the specific underlying molecular mechanism.

**Methods:** Stem cell surface markers, aldehyde dehydrogenase (ALDH) activity, migration and sphere formation abilities were tested to evaluate the stemness of GC cells. PD-L1-expressing lentivirus and PD-L1 specific siRNA were used to analyze the effects of PD-L1 on GC cells stemness. Annexin V/PI double staining was used to assess apoptosis of GC cells induced by chemotherapy. Co-Immunoprecipitation (Co-IP) and Mass spectrometry were employed to determine the PD-L1 binding partner in GC cells. PD-L1^Negative^ and PD-L1^Positive^ cells were sorted by flow cytometry and used for limiting dilution assays to verify the effect of PD-L1 on tumorigenic ability in GC cells.

**Results:** The results showed that GCMSCs enhanced the CSC-like properties of GC cells through PD-L1, which led to the resistance of GC cells to chemotherapy. PD-L1 associated with CTCF to contribute to the stemness and self-renewal of GC cells. *In vivo*, PD-L1^Positive^ GC cells had greater stemness potential and tumorigenicity than PD-L1^Negative^ GC cells. The results also indicated that GC cells were heterogeneous, and that PD-L1 in GC cells had different reactivity to GCMSCs.

**Conclusions:** Overall, our data indicated that GCMSCs enriched CSC-like cells in GC cells, which gives a new insight into the mechanism of GCMSCs prompting GC progression and provides a potential combined therapeutic target.

## Introduction

Gastric cancer (GC) is one of the most common malignancies and the second leading cause of cancer-related deaths wordwide 1. Despite remarkable achievements in surgery, radiotherapy, chemotherapy, and other treatments, GC patients still have a poor 5-year survival rate 2. A better understanding of the molecular mechanisms that underlie GC tumorigenesis is important for improving GC patient outcome. Although cancer stem cells (CSCs) are a very small subset of cancer cells, they are believed to contribute to tumor initiation, heterogeneity, propagation, and therapeutic resistance due to their abilities of self-renewal and multidirectional differentiation 3-6. It has been reported that targeting CSCs could sensitize GC cells to chemotherapy and overcome GC drug resistance 7, 8. CSCs that reside in microenvironmental niches can also escape from the effects of cytotoxic treatments and drive tumor recurrence 9. Epithelial-to-mesenchymal transition (EMT) of cancer cells is associated with the maintenance of stemness properties and other phenotypes of CSCs 10. Biomarkers of GC CSCs were characterized over the past few years, and while this work is not complete. CD44 expression and aldehyde dehydrogenase (ALDH) activity have been recognized as characteristics that can be used to isolate CSCs regardless of the histological classification of GC 11.

Along with tumor cells, the tumor microenvironment (TME) also contains endothelial cells, fibroblasts, immune cells and mesenchymal stem cells (MSCs). Among them, MSCs are a relevant cell type for treatment because of their ability to modulate immune responses, participate in angiogenesis, and promote tumor growth, EMT, and metastasis 12. Our previous studies revealed that MSCs accelerated tumor progression and primarily via the paracrine secretion of soluble cytokines or exosomes 13-15. It is noteworthy that tumor associated MSCs have an important role in modulating the sensitivity of tumor cells to chemotherapy by producing various factors, such as platelet-derived growth factor-C (PDGF-C) 16, hepatocyte growth factor (HGF) 17 and IL‑17A 18. It has also been reported that GCMSCs contributed to GC formation and progression 19. Additionally, we found that GCMSCs exhibited broad immunosuppressive potential, which induced the expression of programmed cell death ligand 1 (PD-L1) in GC cells through the secretion of IL-8 20. IL-8 also played an important role in tumor progression and metastasis by regulating CSCs proliferation and self-renewal 21. The interaction between PD-L1 and its receptor, programmed cell death protein 1 (PD-1) negatively regulates T-cell-mediated immune responses and is associated with resistance to anticancer therapies and poor prognosis 22, 23. Recent studies have indicated that PD-L1 has crucial functions in tumor immune escape, and regulating EMT and CSC-like phenotypes in melanoma and ovarian cancer 24. However, the specific molecular mechanism that regulates the enrichment of CSCs in GC remains unclear.

In this study, we aimed to investigate whether GCMSCs maintained the CSCs pool by up-regulating PD-L1 expression in GC cells, and further explored the underlying molecular mechanism. The results showed that GCMSCs up-regulated the levels of PD-L1 bound to the transcription factor CCCTC-binding factor (CTCF), enhanced the CSC-like properties of GC cells, and led to tumorigenesis. In summary, our data indicated that inhibiting PD-L1 in GC cells may reduce the accumulation of CSC-like cells and alleviate therapeutic resistance in GC patients.

## Materials and Methods

### MSCs and GC cell lines

GC tissues were obtained from GC patients treated at the Affiliated People's Hospital of Jiangsu University (Zhenjiang, Jiangsu, China). The study protocol was approved by the Ethics Committee of Jiangsu University. Informed consent forms were obtained from all subjects. GCMSCs were isolated from human GC tissues as previously described 25. Briefly, fresh GC tissues were cut into approximately 1 mm^3^-sized pieces, which were then adhered to 60 mm cell culture dishes (Corning, USA) and were cultured in MEM-ALPHA (Biological Industries, Israel) supplemented with 10% fetal bovine serum (FBS, Biological Industries) at 37°C with 5% CO_2._ The culture medium was replaced every 3 days. When the fibroblast-like cells reached 85% confluence, the cells were trypsinized and expanded for up to five passages.

The human GC cell lines SGC-7901, MGC-803, HGC-27, and AGS were obtained from the Chinese Academy of Sciences Type Culture Collection Committee Cell Bank (Shanghai, China). SGC-7901, MGC-803, and HGC-27 were cultured in RPMI-1640 (Biological Industries) with 10% FBS. AGS were cultured in DMEM/F-12 (Biological Industries) with 10% FBS at 37°C with 5% CO_2._

### Preparation of GCMSCs conditioned medium (GCMSC-CM)

When the confluence of GCMSCs reached 70%, the culture medium was refreshed and harvested after 48 h. Then the conditioned medium was centrifuged at 1,000 g for 5 min, and then filtered through a 0.22 μm membrane (Millipore, Germany) and stored at -80°C until use.

### Western blot

Western blot was performed using the following antibodies from Cell Signaling Technology (CST): anti-CD44, anti-Sall4, anti-Nanog, anti-Oct4, anti-E-cadherin, anti-N-cadherin, anti-Sox2, anti-PD-L1, anti-Vimentin, anti-CTCF, anti-GAPDH and HRP-conjugated secondary antibody. Signals were detected with ECL reagents (Millipore).

### Migration assay

GC cells were diluted in 200 μL of serum-free RPMI-1640 and seeded into the upper chamber of Transwell assay filters with 8 μm pores (Corning). Then 600 μL of RPMI-1640 supplemented with 10% FBS was added to the lower chamber. Cells were incubated at 37°C with 5% CO_2._ After 8-12 h incubation, cotton swabs were used to remove the cells that did not migrate. The migrated cells were fixed with 4% paraformaldehyde for 30min, stained with crystal violet and imaged.

### Sphere formation assay

First, 2,000 GC cells were cultured in serum-free RPMI-1640 containing EGF (20 ng/mL, PeproTech, USA), b-FGF (20 ng/mL, PeproTech), and B27 (2%, BD Biosciences, USA) and then plated in six-well ultralow attachment plates (Corning). After 10 d, the formed spheres were imaged using a phase-contrast microscope.

### Flow cytometry

GC cells were prepared as a single cell suspension for staining. For surface staining, the following antibodies were used: PE-PD-L1 (eBioscience, USA) and APC-CD44 (eBioscience). The ALDH activity was assayed using the ALDEFLUOR Kit and following the manufacturer's instructions (Stemcell, Canada). Diethylaminobenzaldehyde (DEAB) was added to each sample as a negative control. For analysis of apoptosis, GC cell lines exposed to 5-fluorouracil (5-FU, Grandpharma, China) or paclitaxel (PTX, Beijing Union Pharm, China) for 24 h were harvested. An Annexin V-FITC and PI apoptosis detection kit (BD Biosciences) was used according to the manufacturer's directions. For the sorting of primary GC cells, fresh tumor tissues of GC patients were minced into small pieces and dissociated by IV collagenase (Solarbio, China) at 37°C for 4 h with mild agitation. Then, the suspension was filtered through a 75 μm cell strainer and centrifuged at 300 g for 5 min. Antibody against Pan-CK (Abcam, England) was used to identify primary GC cells. Data were acquired by a flow cytometer (FACSCalibur, BD Biosciences).

### Immunofluorescence

GC cells were incubated overnight at 4°C with the following antibodies: anti-PD-L1, anti-CD44 (CST). Secondary antibodies were applied for 1 h at 37°C. The cells were stained with Hoechst, and then imaged with a confocal microscope (GE, USA).

### Lentivirus transduction and siRNA transfection

To establish stable PD-L1-overexpressing GC cells, a recombinant lentivirus (pLV-PD-L1-puro-GFP) was established following the manufacturer's instruction (GENE, China). Virus-infected GC cells were then selected with puromycin. GC cells were transfected with PD-L1 or CTCF specific siRNAs following the manufacturer's protocol (Genepharma, China).

### Quantitative real-time reverse transcription-polymerase chain reaction (qRT-PCR)

GC cells were lysed with TRIzol reagent (Invitrogen, USA) and to extract total RNA. cDNA was synthesized with qRT-PCR Kits (CWBIO, China). qRT-PCR were performed using the UltraSYBR Mixture (CWBIO). The sequences for sense and antisense primers were as follows: 5′-TCACTTGGTAATTCTGGGAGC-3′ (PD-L1 forward), 5′-CTTTGAGTTTGTATCTTGGATGCC-3′ (PD-L1 reverse); 5′-GCAAAGACCTGTACGCCAACA-3′ (β-actin forward), 5′-TGCATCCTGTCGGCAATG-3′ (β-actin reverse).

### Co-immunoprecipitation (Co-IP)

GC cells were lysed in RIPA buffer with protease inhibitor cocktail (cOmplet, EDTA-free, Roche). After removing cellular debris by centrifugation, the extracts were incubated overnight with anti-PD-L1 antibody (1:50, CST) or IgG (1 μg/mL, CST) at 4°C. Then protein A/G PLUS-Agarose (Santa Cruz, USA) were added and incubated for 4 h at 4°C. Proteins eluted from the protein A/G beads were detected by western blot. Mass spectrometry analysis of protein bands was performed at Shanghai Applied Protein Technology Co. Ltd (China). To detect the interaction between PD-L1 and CTCF, anti-PD-L1 antibody and anti-CTCF antibody (4 μg/mL, Abcam) were used.

### Immunohistochemistry (IHC)

GC sections were obtained from formalin-fixed paraffin-embedded tissues from GC patients. Then GC sections were dewaxed in xylene, rehydrated with an ethanol gradient, and treated in citrate buffer for antigen retrieval. Samples were stained with following antibodies: anti-PD-L1, anti-CD44, and anti-CTCF at 4°C overnight, followed by incubation with secondary biotinylated antibody for 30 min at 37°C. Finally, the slides were visualized with DAB solution and counterstained with hematoxylin. Each stained sample was evaluated by three senior pathologists and five sights were typically selected.

### Tumor formation *in vivo*

SGC-7901 were treated with GCMSC-CM. The PD-L1^Positive^ cells were sorted according to isotype control, as were the same proportions of PD-L1^Negative^ cells. Serial numbers (7×10^2^, 7×10^3^, 7×10^4^ and 7×10^5^) of sorted GC cells were subcutaneously injected into each flank of five-week-old male BALB/c nude mice. Tumor incidence was monitored within 20 d after injection. The tumor tissues were harvested and used to detect the levels of stemness markers.

### Statistical analyses

Data are expressed as mean ± standard deviation (SD). Significance was tested by the Student's t test, ANOVA test, or Kruskal-Wallis H test. The correlation between IL-8 expression and PD-L1 expression in GC patients was analyzed using Spearman's correlation test. *P*< 0.05 was considered to be statistically significant.

## Results

### GCMSCs derived IL-8 enhances the CSC-like properties of GC cells

**Figure 1:**
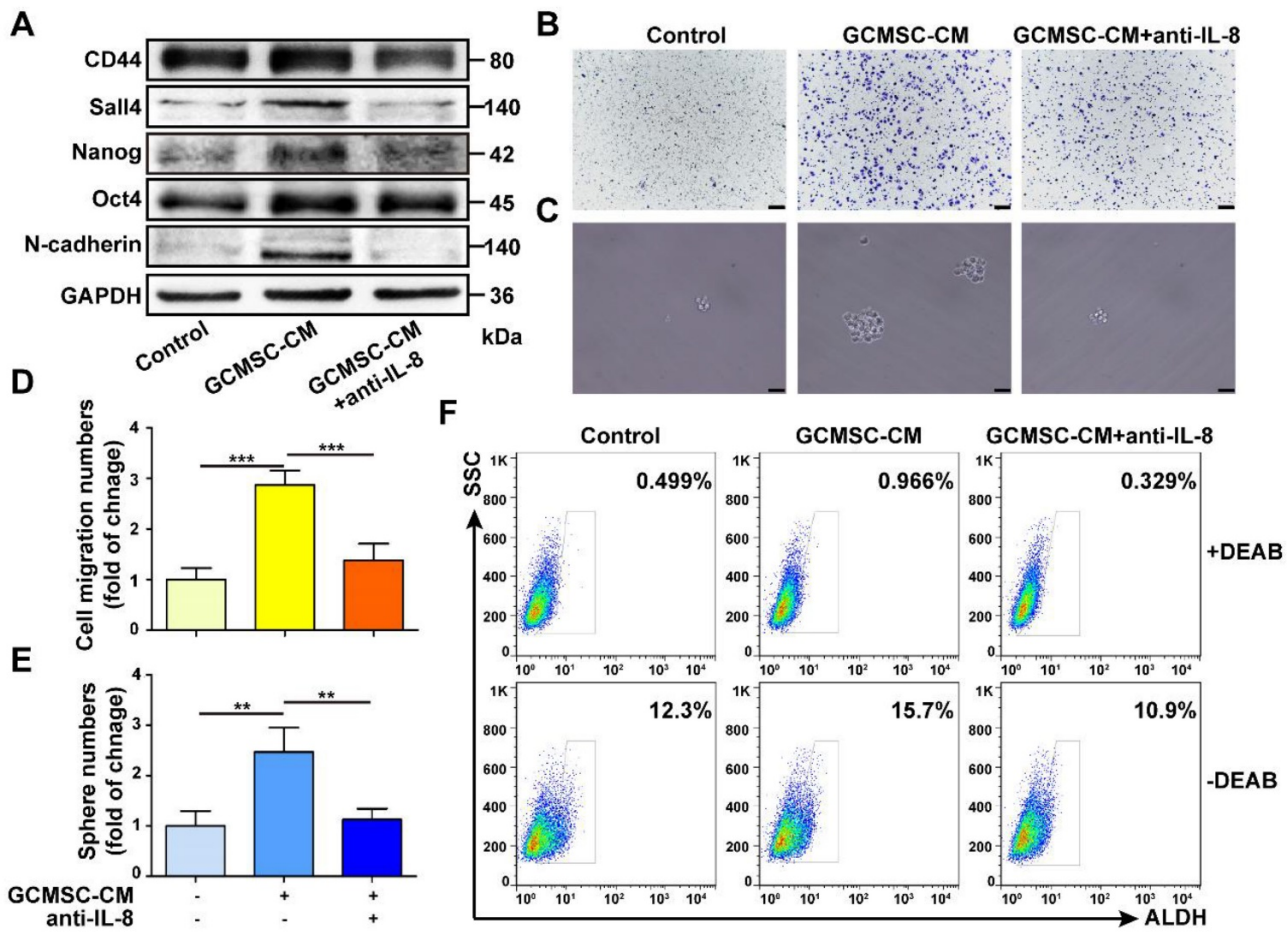
** IL-8 derived from GCMSCs promotes the stemness of GC cells. (A)** Levels of CD44, Sall4, Nanog, Oct4, and N-cadherin in SGC-7901 were examined by western blot.** (B, C)** Transwell migration (scale bar, 100 μm) and sphere formation (scale bar, 50 μm) assays were performed in SGC-7901 following GCMSC-CM treatment.** (D, E)** Quantification of cell migration and sphere numbers.** (F)** The ALDH activity of SGC-7901 was tested by ALDEFLUOR analyses. An IL-8 neutralizing antibody was added to GCMSC-CM and incubated at room temperature for 1 h earlier. The concentration of IL-8 neutralizing antibody was 5 μg/mL (R&D Systems). Data in D and E represents the mean ± SD of three repeated experiments (n=3). GCMSCs were isolated from three different GC patients. **, *P*<0.01, ***, *P*<0.001.

We found that GCMSC-CM increased the expression of the stemness markers CD44, Sall4, Nanog, Oct4 and of the EMT marker N-cadherin in SGC-7901. Additionally, the migration and sphere formation abilities of SGC-7901 were enhanced following GCMSC-CM treatment. However, these changes were reversed by depleting IL-8 in GCMSC-CM (Figure 1A-E). Besides, GCMSC-CM promoted the ALDH activity in GC cells, which was an assessment criterion for CSCs. The ALDH activity of GC cells decreased when IL-8 neutralizing antibody was added to GCMSC-CM (Figure 1F).

In our previous study, we found that GCMSCs secreted IL-8 induced the expression of PD-L1 in GC cells. Thus, IL-8 expression was detected and assessed in PD-L1^Negative^ and PD-L1^Positive^ tumor tissues of GC patients. The results showed that compared with PD-L1^Negative^ GC tissues, PD-L1^Positive^ GC tissues had higher IL-8 levels (*P*<0.05) (Figure S1A). Serum levels of sPD-L1 were also positively correlated with IL-8 levels in GC patients (*P*<0.01, *R*=0.406) (Figure S1B). RNA-Seq data from The Cancer Genome Atlas (TCGA) of 415 GC patients confirmed these findings: PD-L1 levels were positively correlated with IL-8 levels in GC tissues (*P*<0.001, *R*=0.324) (Figure S1C).

### PD-L1 blockade weakens the ability of GCMSC-CM to enhance the stemness of GC cells

**Figure 2:**
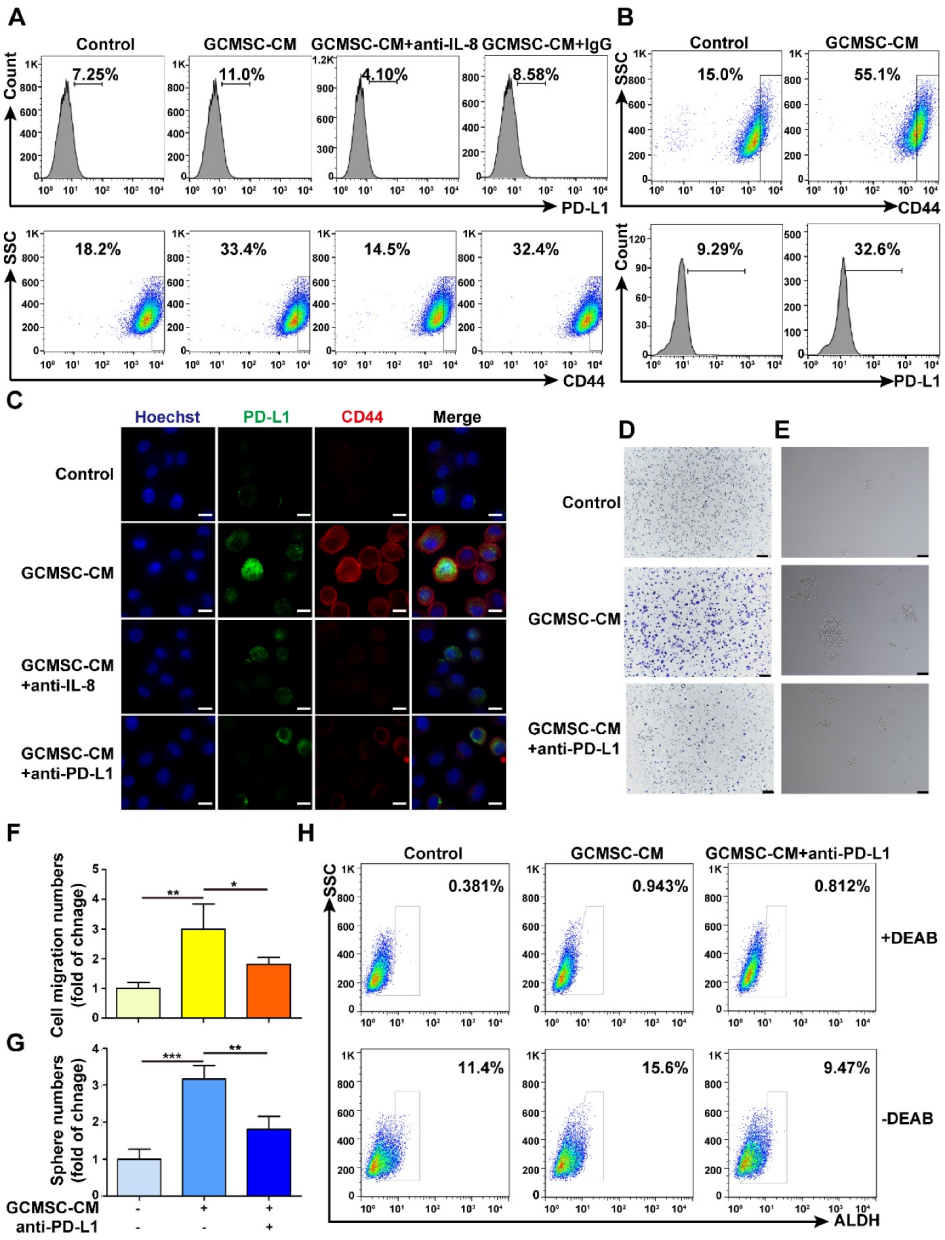
** PD-L1 levels are associated with the effects of GCMSC-CM on increasing the stemness of GC cells. (A)** Levels of PD-L1 and CD44 in SGC-7901 were detected by flow cytometry. **(B)** Levels of PD-L1 in CD44^high^ cells were tested by flow cytometry.** (C)** PD-L1 and CD44 expression in SGC-7901 were detected by immunofluorescence (scale bar, 10 μm).** (D, E)** Transwell migration and sphere formation assays were performed using SGC-7901 following GCMSC-CM treatment (scale bar, 100 μm). **(F, G)** Quantification of cell migration and sphere numbers.** (H)** The ALDH activity of SGC-7901 was tested by ALDEFLUOR analyses. The concentration of anti-PD-L1 neutralizing antibody was 2 μg/mL (eBioscience). Data in F and G represents the mean ± SD of three repeated experiments (n=3). GCMSCs were isolated from three different GC patients. *, *P*<0.05, **, *P*<0.01, ***, *P*<0.001.

Along with the increased expression level of PD-L1 in GC cells that were treated with GCMSC-CM, there was also an increase in CD44^high^ cells increased (Figure 2A). Moreover, PD-L1 was also highly expressed by CD44^high^ cells (Figure 2B). The results showed that GCMSC-CM promoted the expression of PD-L1 and CD44 in SGC-7901, and that this effect was impeded by both depleting IL-8 in GCMSC-CM and by PD-L1 blockade in GC cells (Figure 2C, Figure S2A, B). Meanwhile, we found that GCMSC-CM not only increased PD-L1 expression in the membrane and cytoplasm but also increased nuclear PD-L1 expression (Figure 2C, Figure S2C). Finally, GCMSC-CM was applied to SGC-7901 that had been pre-treated with a PD-L1 neutralizing antibody. The results showed that blocking PD-L1 expression in GC cells could reverse the effects of GCMSC-CM on migration, sphere formation, and ALDH activity (Figure 2D-H).

### PD-L1 is important for maintaining CSC-like phenotypes in GC cells

**Figure 3:**
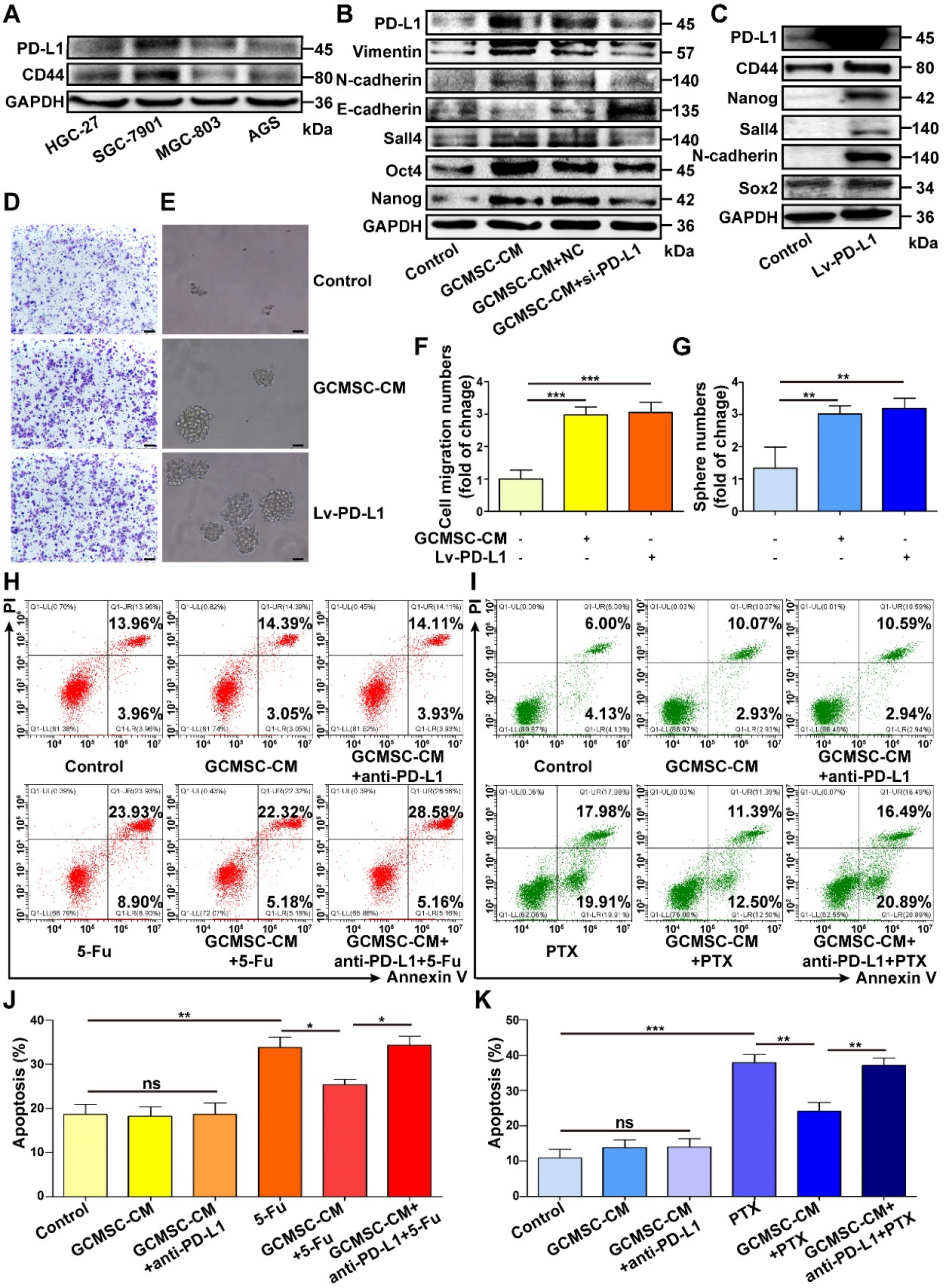
** PD-L1 plays a pivotal role in enhancing the CSC-like properties of GC cells by GCMSCs. (A)** The PD-L1 and CD44 expression of GC cells were tested by western blot. **(B)** The expression of Vimentin, N-cadherin, E-cadherin, Sall4, Oct4, and Nanog in siRNA PD-L1-knockdown SGC-7901 after GCMSC-CM treatment for 24 h were detected by western blot.** (C)** The expression of CD44, Nanog, Sall4, N-cadherin, and Sox2 in HGC-27 transduced with a PD-L1 lentivirus were detected by western blot. **(D, E)** Transwell migration (scale bar, 100 μm) and sphere formation (scale bar, 50 μm) assays were performed in HGC-27 treated with GCMSC-CM or transfected with PD-L1 lentivirus.** (F, G)** Quantification of cell migration and sphere numbers.** (H, I)** Annexin V/PI double staining and flow cytometry were used to assess apoptosis of GC cells induced by chemotherapy. SGC-7901 were exposed to 5-FU for 24 h. HGC-27 were exposed to PTX for 24 h. The concentrations of 5-FU and PTX were 60 μg/mL and 4 ng/mL, respectively. **(J, K)** Quantification of cell apoptosis. Data in F, G, J and K represents the mean ± SD of three repeated experiments (n=3). GCMSCs were isolated from three different GC patients. *, *P*<0.05, **, *P*<0.01, ***, *P*<0.001, ns, not significant. 5-FU, 5-fluorouracil; PTX, paclitaxel.

Next, we detected the expression of PD-L1 and CD44 in different GC cell lines and found that there was a positive correlation between them (Figure 3A). To investigate if PD-L1 expression had a direct role in regulating the stemness of GC cells, we used a PD-L1 specific siRNA to knockdown PD-L1 expression in SGC-7901. The results showed that compared with the GCMSC-CM group, downregulating of PD-L1 significantly reduced the levels of Vimentin, N-cadherin, Sall4, Oct4, and Nanog, but increased the level of E-cadherin levels (Figure 3B). Next, we overexpressed PD-L1 in HGC-27 with a PD-L1-expressing lentivirus. Compared with the control group, the levels of CD44, Nanog, Sall4, N-cadherin, and Sox2 were significantly increased in PD-L1-overexpressing cells (Figure 3C). The migration and sphere formation abilities of HGC-27 cells were enhanced in both the GCMSC-CM group and the PD-L1-overexpressing group (Figure 3D-G). Furthermore, to investigate the effects of GCMSCs on the resistance of GC cells to chemotherapy, GC cells treated with GCMSC-CM were exposed to 5-FU or PTX, and apoptosis was tested. The results showed that the number of apoptotic cells was similar in each group without chemotherapeutic agents. However, when treated with the same concentration of 5-FU, the number of apoptotic cells in the GCMSC-CM group was significantly lower than that in the Control group, and the effect of GCMSC-CM was largely reduced after blocking PD-L1 in GC cells (Figure 3H, J). Similar results were obtained when GC cells exposed to PTX (Figure 3I, K).

### PD-L1^Negative^ and PD-L1^Positive^ GC cells show different reactivity to GCMSCs

**Figure 4:**
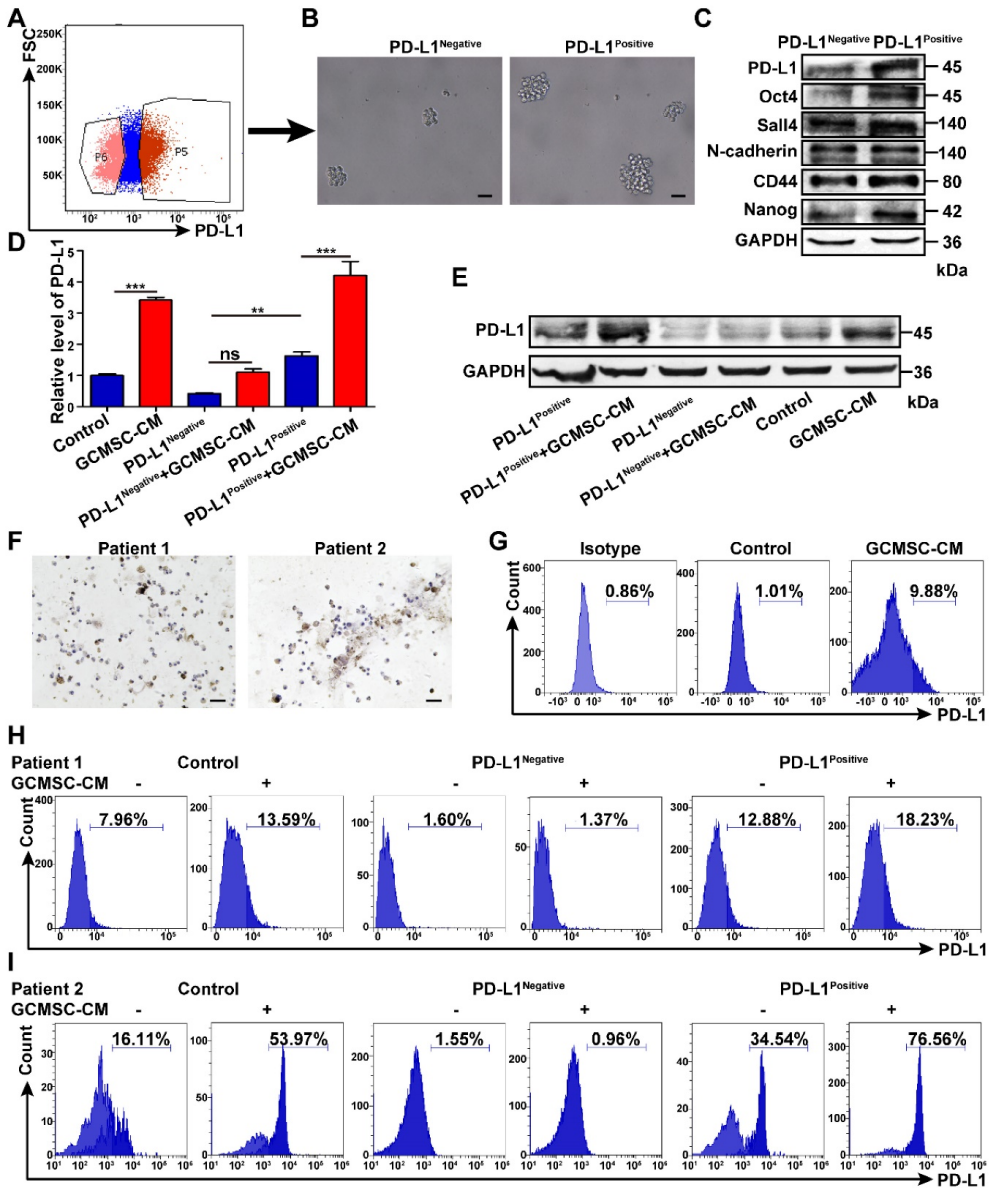
** The responses of PD-L1^Negative^ and PD-L1^Positive^ GC cells to GCMSCs are different. (A)** SGC-7901 were treated with GCMSC-CM for 24 h and then the PD-L1^Negative^ and PD-L1^Positive^ cells were sorted by flow cytometry. **(B, C)** These cells were then used to perform sphere formation assays, and the levels of Oct4, Sall4, N-cadherin, CD44, and Nanog in sphere cells were detected by western blot (scale bar, 50 μm). **(D, E)** The sorted cells were retreated with GCMSC-CM, and the PD-L1 expression was detected by qRT-PCR and western blot.** (F)** Representative images of primary GC cells stained for Pan-CK isolated from GC tissues of 2 GC patients (scale bar, 25 μm). **(G)** Primary GC cells were treated with GCMSC-CM for 24 h and the level of PD-L1 was detected by flow cytometry.** (H, I)** The PD-L1^Negative^ and PD-L1^Positive^ cells in primary GC cells were sorted by flow cytometry and then, treated with GCMSC-CM and the PD-L1 level was detected by flow cytometry. Data in D represents the mean ± SD of three repeated experiments (n=3). **, *P*<0.01, ***, *P*<0.001, ns, not significant.

SGC-7901 were treated with GCMSC-CM and then, PD-L1^Negative^ and PD-L1^Positive^ cells were sorted (Figure 4A). The results showed that the sphere formation ability of PD-L1^Positive^ cells was higher than that of PD-L1^Negative^ cells (Figure 4B). The levels of Oct4, Sall4, N-cadherin, CD44, and Nanog in PD-L1^Positive^ sphere cells were also higher than those of PD-L1^Negative^ sphere cells (Figure 4C). Then, the sorted cells were treated with GCMSC-CM again. The results showed that PD-L1 levels were further increased in PD-L1^Positive^ cells after retreatment with GCMSC-CM. However, the PD-L1 levels remained low in PD-L1^Negative^ cells after retreatment with GCMSC-CM (Figure 4D, E).

Considering the biological variety of GC, primary GC cells were isolated from GC tissues of GC patients (Figure 4F). The results showed that the expression of PD-L1 in primary GC cells treated with GCMSC-CM was increased (Figure 4G). Next, PD-L1^Positive^ and PD-L1^Negative^ cells in primary GC cells were sorted and treated with GCMSC-CM for 24 h. The results showed that the level of PD-L1 in PD-L1^Positive^ primary GC cells from different GC tissues further increased to different degrees after treatment with GCMSC-CM; However, the PD-L1 level remained low in PD-L1^Negative^ primary GC cells after treatment with GCMSC-CM (Figure 4H, I). The results were consistent in different GC primary cultures and GC cell lines.

### PD-L1^Positive^ subpopulation of GC cells possesses increased tumorigenicity

**Figure 5:**
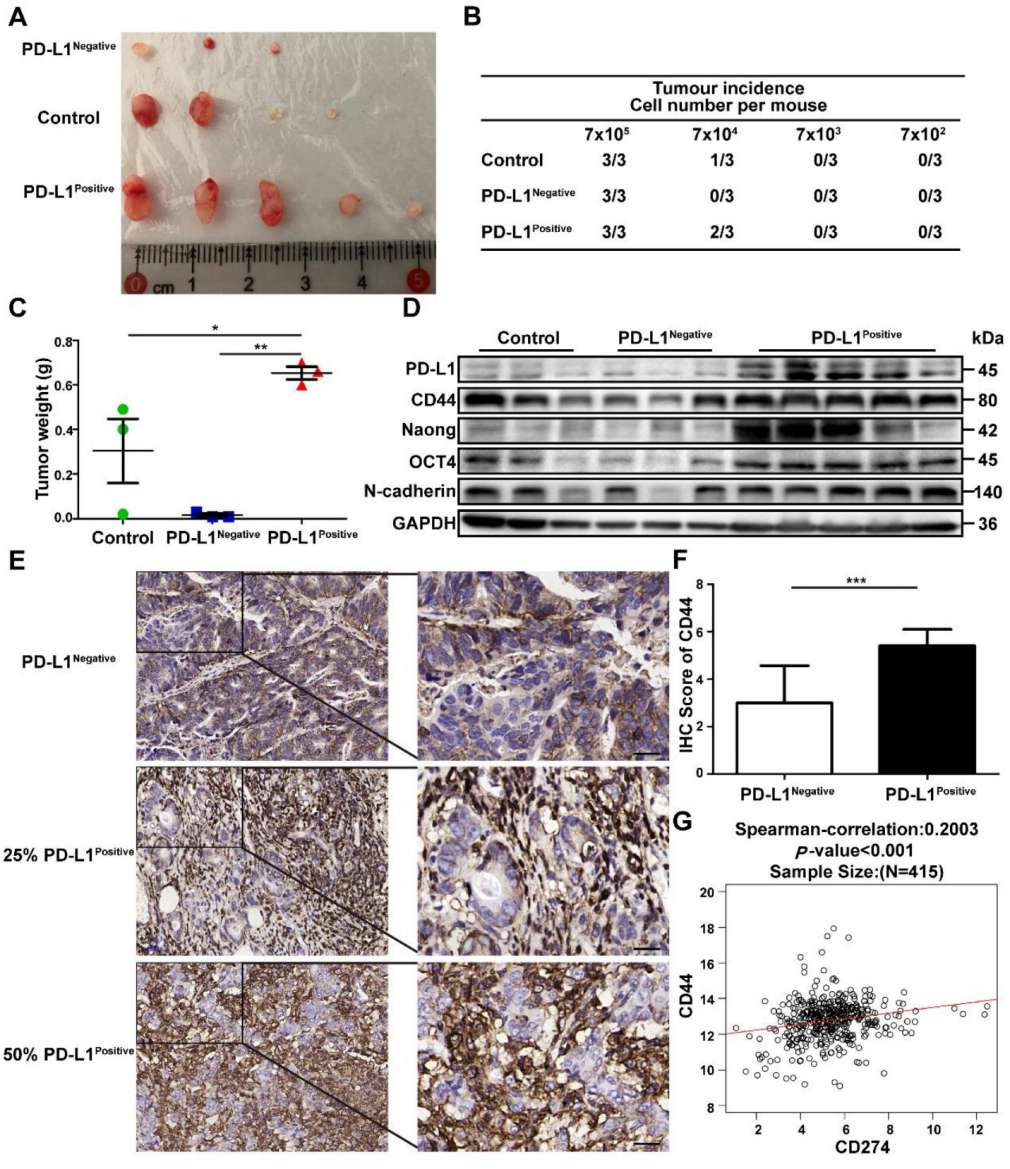
** The PD-L1^Positive^ subpopulation of GC cells has higher tumor-initiation potential.** SGC-7901 were treated with GCMSC-CM, and then the PD-L1^Negative^ and PD-L1^Positive^ cells were sorted by flow cytometry, counted, and injected subcutaneously in limiting dilution assays into BALB/c nude mice. After 20 d, the mice were sacrificed and the tumor tissues were collected.** (A)** Representative images of tumors from the indicated group were shown.** (B)** The table displays the number of mice that developed tumors. **(C)** Tumors Weights in mice on day 20 after injection with 7×10^5^ sorted GC cells (n=3). *, *P*<0.05, **, *P*<0.01.** (D)** The levels of PD-L1, CTCF, CD44, Nanog, Oct4, and N-cadherin in tumor tissues were detected by western blot. **(E, F)** Representative IHC staining and quantification of the CD44 levels in PD-L1^Negative^ and PD-L1^Positive^ tumor tissues from GC patients (scale bar, 50 μm, n=20). ***, *P*<0.001. (G) Correlations between PD-L1 and CD44 in GC tissues from 415 patients in TCGA data set. ***, *P*<0.001.

SGC-7901 were treated with GCMSC-CM, and then PD-L1^Negative^ and PD-L1^Positive^ cells were sorted by flow cytometry, counted, and injected subcutaneously in limiting dilution assays into BALB/c nude mice. The results showed that PD-L1^Positive^ cells possessed higher tumor-initiation rates, driving greater tumor growth potential than untreated cells and PD-L1^Negative^ cells (Figure 5A-C). Furthermore, we detected the stemness markers in different tumor tissues. We found that the levels of PD-L1, CD44, Nanog, Oct4, and N-cadherin were higher in the PD-L1^Positive^ group than in the control and PD-L1^Negative^ groups (Figure 5D). Next, CD44 expression was detected and assessed in PD-L1^Negative^ and PD-L1^Positive^ tumor tissues from GC patients. The results showed that compared with PD-L1^Negative^ GC tissues, CD44 levels were higher in PD-L1^Positive^ GC tissues (*P*<0.001) (Figure 5E, F). TCGA RNA-Seq data of 415 GC patients confirmed these results: the PD-L1 levels were positively correlated with CD44 levels in GC tissues (*P*<0.001, *R*=0.2003) (Figure 5G).

### PD-L1 associates with CTCF to contribute to the stemness and self-renewal of GC cells

**Figure 6:**
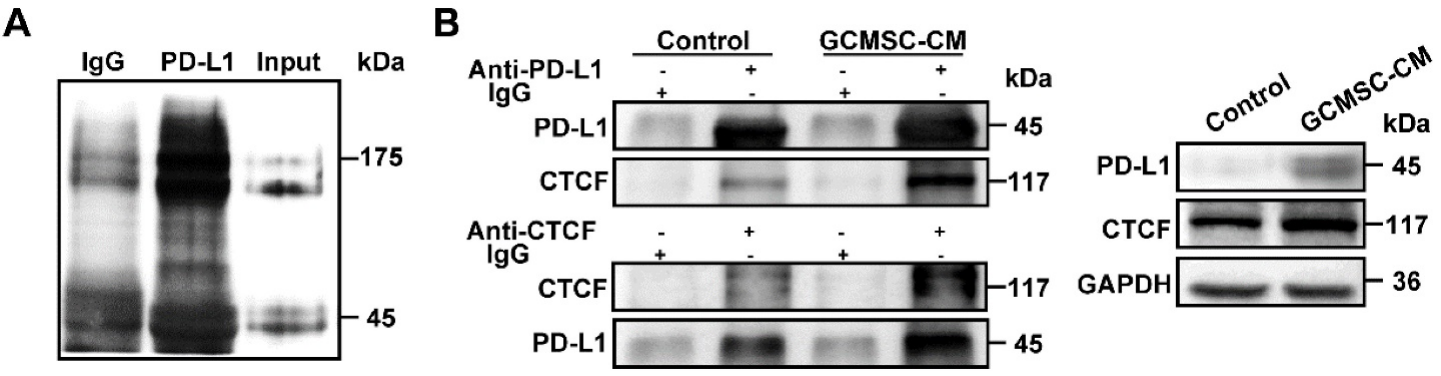
** CTCF is a PD-L1 binding partner in GC cells. (A)** Anti-PD-L1 antibody, but not IgG, coprecipitated a protein band in SGC-7901.** (B)** Co-IP assay identified an association of CTCF with PD-L1 in SGC-7901. Whole cell lysate was used as input. The association of CTCF with PD-L1 was using anti-PD-L1 antibody or anti-CTCF antibody.

**Figure 7:**
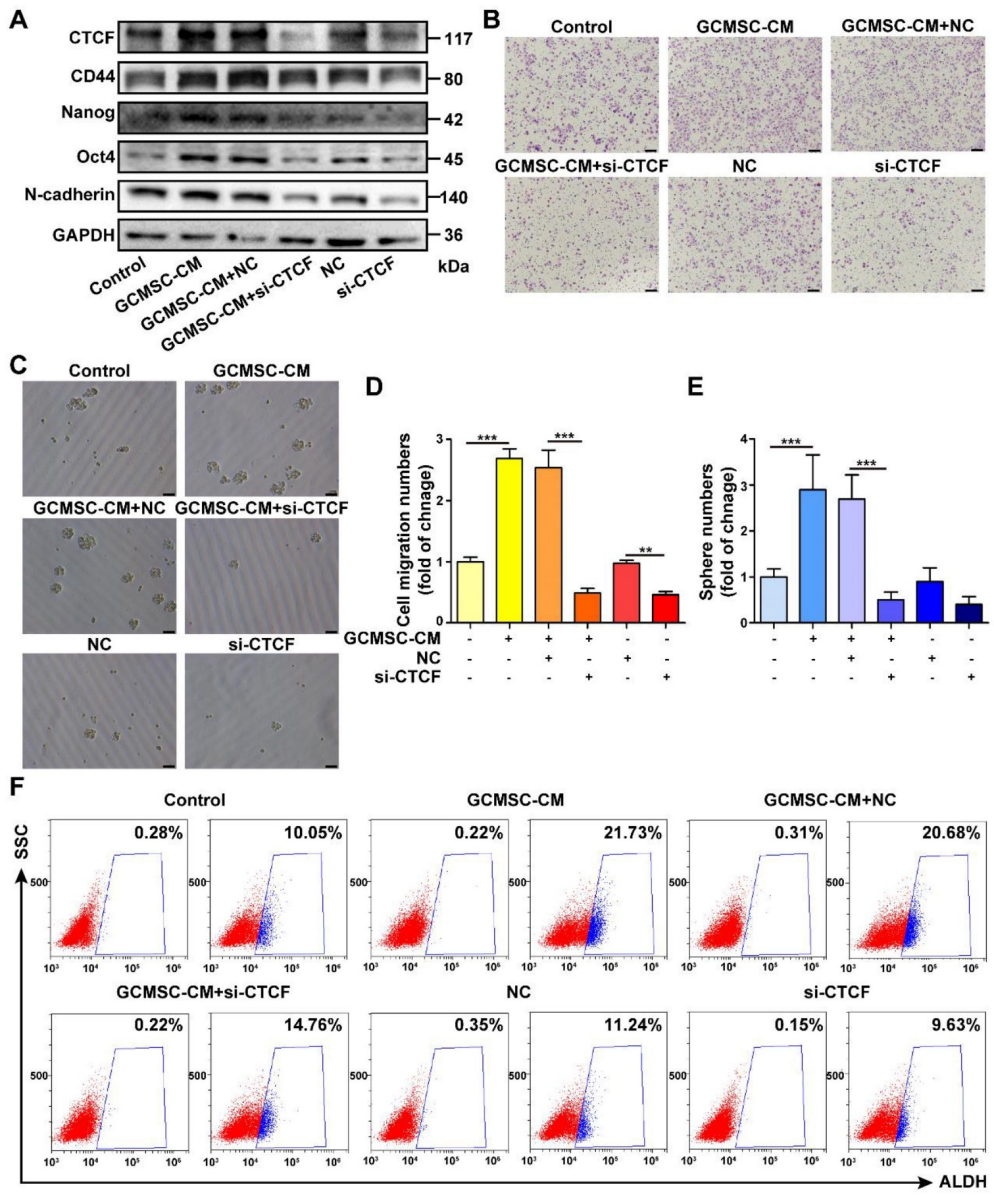
** PD-L1 enhances the CSC-like properties of GC cells via binding CTCF. (A)** The expression of CD44, Nanog, Oct4, and N-cadherin in SGC-7901 following siRNA mediated knockdown of CTCF and GCMSC-CM treatment for 24 h were detected by western blot. **(B, C)** Transwell migration (scale bar, 100 μm) and sphere formation (scale bar, 50 μm) assays were performed in siRNA CTCF-knockdown SGC-7901 following GCMSC-CM treatment for 24 h.** (D, E)** Quantification of cell migration and sphere numbers. **(F)** The ALDH activity of siRNA CTCF-knockdown SGC-7901 following GCMSC-CM treatment for 24 h was tested by ALDEFLUOR analyses. Data in D and E represents the mean ± SD of three repeated experiments (n=3). GCMSCs were isolated from three different GC patients. **, *P*<0.01, ***, *P*<0.001.

To identify a potential intracellular, functional partner of PD-L1, we performed IP assays using an anti-PD-L1 antibody. We identified a 175 kDa protein band that coprecipitated with PD-L1 in SGC-7901 (Figure 6A). Mass spectrometry analyses indicated that several proteins were pulled-down with PD-L1, among which, we selected CTCF, which is stemness-related and involved in tumor cell self-renewal. To further validate the interaction between PD-L1 and CTCF, we performed Co-IP assays. The results showed that PD-L1 and CTCF were mutually pulled down by their respective antibodies using SGC-7901 lysate (Figure 6B). Then, we used a CTCF specific siRNA to knockdown CTCF expression in GC cells. The results showed that compared with the GCMSC-CM group, silencing CTCF significantly decreased the levels of CD44, Nanog, Oct4, and N-cadherin (Figure 7A). Similarly, silencing CTCF reduced the migration ability of GC cells (Figure 7B, D). The effects of CTCF on regulating the expression of stemness markers and migration ability were also detected in HGC-27 (Figure S3A-C). These results also showed that silencing CTCF in SGC-7901 could reverse the effects of GCMSC-CM on promoting sphere formation (Figure 7C, E) and increasing ALDH activity (Figure 7F).

## Discussion

GC is one of the most common malignancies worldwide and currently accounts for 8.2% of all new cancer cases. Despite significant progress in detection and therapeutic strategies over the past decade, the 5-year survival rate of GC patients remains low 26, 27. CSCs are a very small subpopulation of cancer cells residing in TME and are believed to contribute to tumor initiation, heterogeneity, propagation, and therapeutic resistance owing to their abilities of self-renewal and multidirectional differentiation 28-30. Recently, Zhao *et al.* reported that miR-6778-5p strengthened CSCs stemness via regulating of cytosolic one-carbon folate metabolism 31. However, the specific mechanism of inducing CSCs enrichment in GC is poorly understood.

Over the past few years, MSCs have attracted extensive research attention because of their capacities to influence the occurrence and development of tumors 32-35. In this study, GCMSCs used in independent experiments were from different GC patients. Our results showed that GCMSC-CM promoted the expression of stemness markers, increased migration and sphere formation abilities, and enhanced ALDH activity in GC cells. Together, these data indicated that GCMSC-CM enhanced the CSC-like properties of GC cells. It has been reported that PD-L1 overexpression can affect the therapeutic efficacy of chemotherapy and shorten the survival period of patients 36, 37. The results showed that GCMSCs promoted the resistance of GC cells to chemotherapy. However, the sensitivity of GC cells to chemotherapy was enhanced when PD-L1 was blocked.

Hsu *et al*. proved that PD-L1 expression in CSCs was abundant which contributed to immune evasion 38. Our previous studies found that GCMSCs derived IL-8 induced the expression of PD-L1 in GC cells 20. So here we asked whether GCMSCs maintained the CSCs pool by up-regulating PD-L1. Immunofluorescence results showed that GCMSC-CM not only up-regulated membrane and cytoplasmic PD-L1 expression but also increased the nuclear fraction of PD-L1. Satelli *et al*. showed that nuclear PD-L1 levels of circulating tumor cells were significantly associated with shorter survival in metastatic colorectal cancer patients and metastatic prostate cancer patients 39. It has also been reported that tumor cell-intrinsic PD-L1 contributes to cancer stemness, EMT, tumor invasion, and chemoresistance in multiple tumor types 40.

Next, our results showed that when PD-L1 was knocked down with a specific siRNA in GC cells, the effects of GCMSC-CM on increasing the levels of stemness markers, promoting the migration and sphere formation abilities, and enhancing ALDH activity were impeded. To confirm the relationship between PD-L1 and stemness, PD-L1^Negative^ and PD-L1^Positive^ GC cells treated with GCMSC-CM were sorted. Compared with the PD-L1^Negative^ group, PD-L1^Positive^ GC cells had a higher sphere formation capacity. At the same time, limiting dilution assays were performed *in vivo*, and the results showed the stronger tumor-initiation potential of PD-L1^Positive^ GC cells.

It is noteworthy that the reactivity of PD-L1^Positive^ cells and PD-L1^Negative^ cells to GCMSC-CM was different. The PD-L1 level of PD-L1^ Positive^ cells increased with GCMSC-CM treatment in both GC cell lines and primary GC cells; however, unlike PD-L1^Positive^ cells, the level of PD-L1 in PD-L1^Negative^ cells was remained low after treatment with GCMSC-CM. This indicated the heterogeneity of GC cells with regards to PD-L1 expression and reactivity to GCMSC-CM. As for the reason why a subset of GC cells did not respond to GCMSC-CM, we will continue to expand the sample size and explore this in subsequent experiments.

To further investigate how PD-L1 enhanced the CSC-like properties of GC cells, we performed IP assays with an anti-PD-L1 antibody. We identified a 175 kDa protein band that coprecipitated with PD-L1 in GC cells. Combined with Mass spectrometry analyses results and literature reports, CTCF, a candidate tumor suppressor gene that encodes a multifunctional transcriptional factor attracted our attention. Surprisingly for a tumor suppressor, CTCF levels were increased in breast cancer compared with normal breast tissues. The increase in CTCF was also linked to the resistance of breast cancer cells to apoptosis 41. Liu *et al.* found that the promoter region of OCT4 contained CTCF binding sequences and that active OCT4 might directly regulate the downstream target genes SOX2, NANOG, and CD90, further promoting liver CSC-like phenotypes such as self-renewal, migration, invasion, and chemoresistance 42. Zhao *et al.* proved that CTCF targeted the MYCN promoter, resulting in increased MYCN expression, suppressed differentiation, and the promotion of growth, metastasis, and invasion of neuroblastoma cells *in vitro* and *in vivo* 43. Huang *et al.* also indicated oncogenic roles for CTCF in tumorigenesis 44. To further validate the interaction between PD-L1 and CTCF, we performed Co-IP assays. The results showed that PD-L1 and CTCF in GC cells were mutually pulled down by their respective antibodies. Additionally, when CTCF was knocked down by specific siRNA in GC cells, the effects of GCMSC-CM on increasing the levels of stemness markers, promoting the migration and sphere formation abilities, and enhancing ALDH activity were impeded.

In summary, this study showed that GCMSCs increased the level of PD-L1 bound to CTCF, strengthened the CSC-like properties of GC cells, and led to tumorigenesis. Blocking PD-L1 expression in GC cells may inhibit the accumulation of CSC-like cells, providing a potential strategy to alleviate therapeutic resistance in GC patients.

## Supplementary Material

Supplementary figures and tables.
