# Supplementary material for: Gastric cancer mesenchymal stem cells regulate PD-L1-CTCF enhancing cancer stem cell-like properties and tumorigenesis
Source: Theranostics. 2020 Oct 25;10(26):11950–62. doi: 10.7150/thno.49717 (PMC7667687; doi:10.7150/thno.49717)
Supplement: Supplementary file 1 — Supplementary figures and tables. [file thnov10p11950s1.pdf]

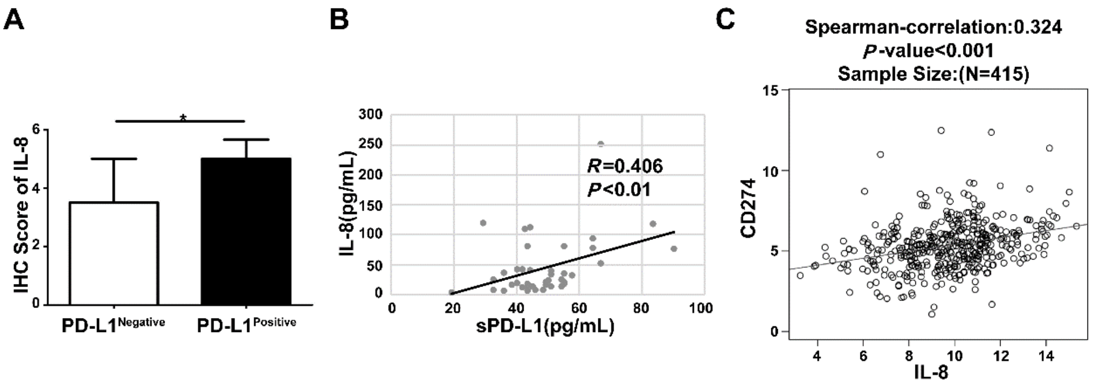

Figure S1. Relationship between IL-8 and PD-L1 levels in GC patients. (A) IHC scores of IL-8 in PD-L1^{Negative} and PD-L1^{Positive} tumor tissues of 20 GC patients. *, *P*<0.05. (B) Correlations between IL-8 and sPD-L1 serum levels of GC patients (n=40). *R*=0.406, **, *P*<0.01. (C) Correlations between IL-8 and PD-L1 in GC tissues from 415 patients in TCGA data set. ***, *P*<0.001.

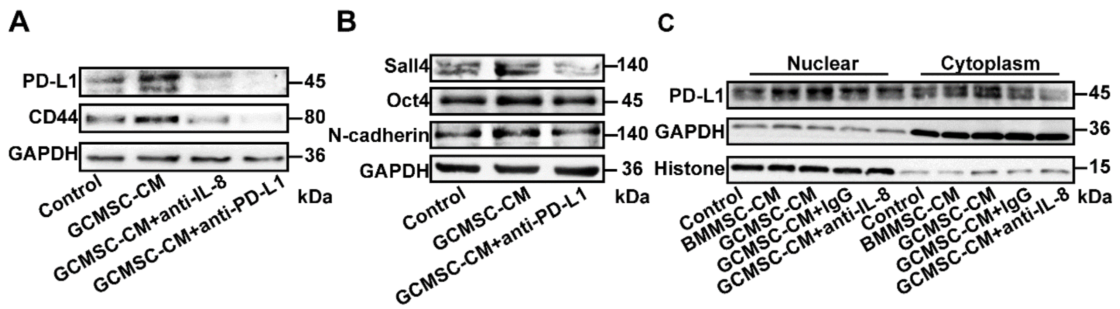

Figure S2. IL-8 secreted by GCMSCs increases the stemness of GC cells through PD-L1. (A) The expression of PD-L1 and CD44 in SGC-7901 treated with GCMSC-CM (with or without IL-8 depletion or PD-L1 blockade) was detected by western blot. (B) The expression of stemness markers in SGC-7901 treated with GCMSC-CM (with or without PD-L1 blockade) was detected by western blot. (C) PD-L1 levels in nuclear and cytoplasm was examined by western blot. BMMSC-CM, Bone marrow MSCs conditioned medium.

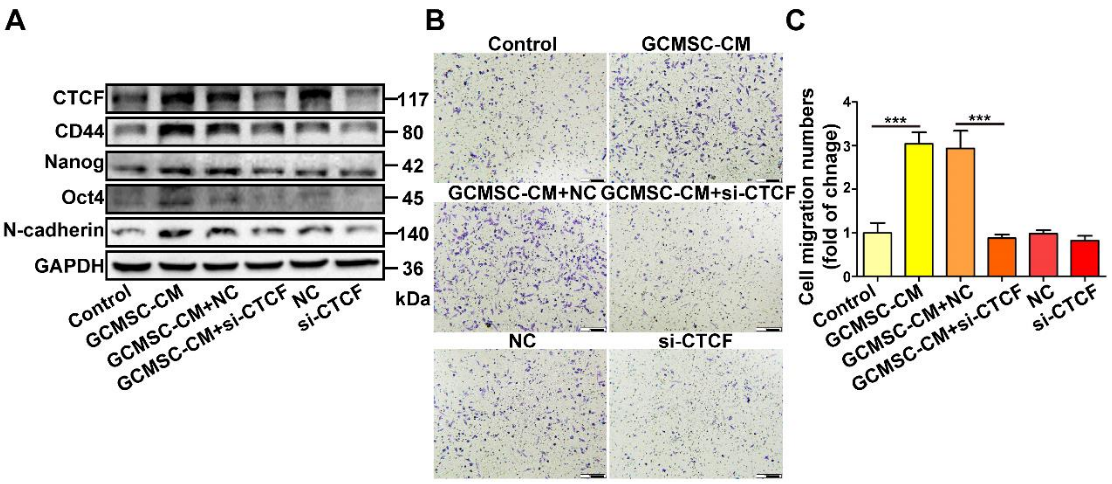

Figure S3. PD-L1 regulates the stemness of GC cells through the association with CTCF. (A) The expression of CD44, Nanog, Oct4, and N-cadherin in siRNA CTCF-knockdown HGC-27 following GCMSC-CM treatment for 24 h were detected by western blot. (B) Transwell migration assays (scale bar, 100 μm) were performed in siRNA CTCF-knockdown HGC-27 following GCMSC-CM treatment for 24 h. (C) Quantification of the cell migration numbers. Data in C represents the mean ± SD of three repeated experiments (n=3). GCMSCs were isolated from three different GC patients. ***, *P*<0.001.
